# Screen for abnormal mitochondrial phenotypes in mouse embryonic stem cells identifies a model for succinyl-CoA ligase deficiency and mtDNA depletion

**DOI:** 10.1242/dmm.013466

**Published:** 2013-11-21

**Authors:** Taraka R. Donti, Carmen Stromberger, Ming Ge, Karen W. Eldin, William J. Craigen, Brett H. Graham

**Affiliations:** 1 Department of Molecular and Human Genetics, Baylor College of Medicine, One Baylor Plaza, Houston, TX 77030, USA.; 2 Department of Pathology and Immunology, Baylor College of Medicine, One Baylor Plaza, Houston, TX 77030, USA.; 3 Department of Pediatrics, Baylor College of Medicine, One Baylor Plaza, Houston, TX 77030, USA.

**Keywords:** TCA cycle, Mitochondrial DNA depletion, Gene trap, Mitochondria

## Abstract

Mutations in subunits of succinyl-CoA synthetase/ligase (SCS), a component of the citric acid cycle, are associated with mitochondrial encephalomyopathy, elevation of methylmalonic acid (MMA), and mitochondrial DNA (mtDNA) depletion. A FACS-based retroviral-mediated gene trap mutagenesis screen in mouse embryonic stem (ES) cells for abnormal mitochondrial phenotypes identified a gene trap allele of *Sucla2* (*Sucla2^SAβgeo^*), which was used to generate transgenic mice. *Sucla2* encodes the ADP-specific β-subunit isoform of SCS. *Sucla2^SAβgeo^* homozygotes exhibited recessive lethality, with most mutants dying late in gestation (e18.5). Mutant placenta and embryonic (e17.5) brain, heart and muscle showed varying degrees of mtDNA depletion (20–60%). However, there was no mtDNA depletion in mutant liver, where the gene is not normally expressed. Elevated levels of MMA were observed in embryonic brain. SCS-deficient mouse embryonic fibroblasts (MEFs) demonstrated a 50% reduction in mtDNA content compared with wild-type MEFs. The mtDNA depletion resulted in reduced steady state levels of mtDNA encoded proteins and multiple respiratory chain deficiencies. mtDNA content could be restored by reintroduction of *Sucla2*. This mouse model of SCS deficiency and mtDNA depletion promises to provide insights into the pathogenesis of mitochondrial diseases with mtDNA depletion and into the biology of mtDNA maintenance. In addition, this report demonstrates the power of a genetic screen that combines gene trap mutagenesis and FACS analysis in mouse ES cells to identify mitochondrial phenotypes and to develop animal models of mitochondrial dysfunction.

## INTRODUCTION

Mitochondrial disease is a significant cause of heritable multiorgan dysfunction. Current epidemiological evidence suggests that the prevalence of mitochondrial disorders might be as high as 1 in 5000, making mitochondrial disease one of the more common genetic causes of encephalomyopathies and multisystem disease (Schaefer et al., 2004; Elliott et al., 2008; Schaefer et al., 2008). Despite important insights into clinical, biochemical and molecular features of these disorders, the underlying molecular pathogenesis remains poorly understood and no clearly effective therapies exist. Mitochondria contain their own genome that consists of a multicopy, ~16.4-kilobase circular chromosome. This mitochondrial DNA (mtDNA) encodes 13 polypeptides that are subunits of various respiratory chain complexes as well as 22 tRNAs and two rRNAs required for mitochondrial protein translation. The mitochondrial proteome consists of ~1300 proteins, therefore the remaining 99% of mitochondrial proteins are nuclear encoded, including all of the protein machinery required for mtDNA replication, maintenance, transcription and translation (Calvo and Mootha, 2010). Mitochondrial disease can be caused by mutations in mtDNA or in nuclear-encoded genes, with the majority of pediatric cases of mitochondrial disease presumably caused by recessive mutations in nuclear-encoded genes, of which only a small fraction are identified (Haas et al., 2008).

Mitochondrial encephalomyopathy with mtDNA depletion represents an important subset of mitochondrial diseases and is defined by a global or tissue-specific reduction in mtDNA copy number. Over the last decade, mitochondrial diseases associated with mtDNA depletion have been described and a number of causative genes identified (Graham, 2012). These clinically heterogeneous disorders are autosomal recessive and encompass a wide spectrum of clinical features, including combinations of infantile or childhood encephalopathy with severe intellectual disability, myopathy, cardiomyopathy and hepatopathy. Nuclear-encoded genes associated with mtDNA depletion syndromes include genes important for mtDNA replication (*POLG*, *TWINKLE*), regulation of mitochondrial nucleotide pools (*DGUOK*, *TP*, *TK2*, *RRM2B*) and genes with poorly defined functions related to mtDNA maintenance, including, *MPV17* and, interestingly, subunits of the Kreb’s cycle enzyme succinyl-CoA synthetase, SCS (*SUCLG1*, *SUCLA2*) (Suomalainen and Isohanni, 2010). Although animal models have been reported for many of these genes (Haraguchi et al., 2002; Kimura et al., 2003; Hance et al., 2005; Tyynismaa et al., 2005; Akman et al., 2008; Martínez-Azorín et al., 2008; López et al., 2009; Viscomi et al., 2009), there is currently no reported animal model for SCS-dependent mtDNA depletion.

SCS is the TCA cycle enzyme responsible for the conversion of succinyl-CoA to succinate in the mitochondrial matrix and is coupled to the phosphorylation of GDP or ADP, thereby providing the only ‘substrate level’ phosphorylation in the TCA cycle. SCS is a heterodimer, composed of a catalytic α-subunit (SUCLG1) and a dNDP-binding β-subunit. There are two isoforms of the β-subunit: ADP-specific (SUCLA2) and GDP-specific (SUCLG2) isoforms. Expression studies demonstrate that these isoforms are widely expressed but exhibit differential expression patterns in tissues, with *Sucla2* expressed highest in mouse brain, heart and skeletal muscle and *Suclg2* predominating in liver and kidney (Lambeth et al., 2004). Mutations in *SUCLA2* were first identified as a cause of severe mitochondrial encephalomyopathy with skeletal muscle mtDNA depletion through homozygosity mapping of a consanguineous family with multiple affected members (Elpeleg et al., 2005). Subsequently, it was demonstrated that *SUCLA2*-associated mitochondrial encephalomyopathy with mtDNA depletion is quite common in the Faroe Island population, with an incidence of 1 in 1700 secondary to a founder mutation in *SUCLA2* (Ostergaard et al., 2007b). These patients also exhibit modest elevations of methylmalonic acid (MMA), presumably due to secondary inhibition of methylmalonyl-CoA mutase by accumulation of succinyl-CoA resulting from SCS deficiency (Carrozzo et al., 2007). Mutations in the α-subunit gene of SCS (*SUCLG1*) have also been reported in association with mitochondrial encephalomyopathy with mtDNA depletion in skeletal muscle (Ostergaard et al., 2007a).

**RESOURCE IMPACT**

**Background**

Mitochondrial disease associated with loss of cellular mitochondrial DNA content (mtDNA depletion) is characterized by a global or tissue-specific reduction in mtDNA copy number. Mitochondrial disease with mtDNA depletion can be caused by mutations in one of several genes and can cause dysfunction of one or more organs, including brain, heart, skeletal muscle and liver. *SUCLA2* is one of these genes and encodes the ADP-specific β-subunit of succinyl-CoA synthetase (SCS), an enzyme responsible for conversion of succinyl-CoA to succinate in the Krebs (citric acid) cycle. Patients with *SUCLA2* mutations generally exhibit intellectual disability, severe low muscle tone, dystonia and deafness. Mild elevation of methylmalonic acid (MMA) and loss of mtDNA in muscle are considered hallmarks of *SUCLA2* deficiency. Currently, animal models for *SUCLA2* deficiency are lacking, the underlying disease mechanisms are poorly understood and no efficacious treatments are available.

**Results**

By performing a FACS-based retroviral-mediated gene trap mutagenesis screen designed to detect abnormal mitochondrial phenotypes in mouse embryonic stem (ES) cells, the authors isolated a mutant allele of *Sucla2*, and these mutant ES cells were used to generate transgenic mice. Animals deficient for *Sucla2* exhibited embryonic lethality with the mutant embryos dying late in gestation. Histological analysis of mutant placenta revealed increased mineralization and mutant embryos were found to be approximately 25% smaller than wild-type littermates. *Sucla2* mutant placenta as well as mutant embryonic brain, heart and skeletal muscle showed varying degrees of mtDNA depletion and mutant brains exhibited elevated levels of MMA. SCS-deficient mouse embryonic fibroblasts (MEFs) demonstrated a 50% reduction in mtDNA content compared with normal MEFs. The mtDNA depletion in MEFs and embryonic tissues was revealed to be functionally significant, as it resulted in reduction of steady state levels of mtDNA-encoded proteins, multiple respiratory chain deficiencies, and cellular respiration defects. Furthermore, mtDNA content was restored in mutant cells by reintroduction of *Sucla2*.

**Implications and future directions**

The demonstration of SCS deficiency, mtDNA depletion and elevation of MMA validates the *Sucla2* mutant mouse as a model for *SUCLA2*-dependent mitochondrial disease with mtDNA depletion. Future studies of this model of SCS deficiency will provide further insights into the pathogenesis of this category of mitochondrial diseases and into the biology of mtDNA maintenance, as well as facilitating the exploration of novel therapeutic strategies. The implementation of genetic strategies to bypass embryonic lethality in *Sucla2* mutants should allow the recovery and study of adult animals with global or tissue-specific *Sucla2* deficiency to provide additional insights into disease pathogenesis and mtDNA biology. Finally, the study demonstrates the utility of the FACS-based genetic screen used by the authors to establish novel animal models of mitochondrial biology and disease.

Here, we report the isolation of a mutant allele of *Sucla2* in mouse embryonic stem (ES) cells from a genetic screen designed to identify abnormal mitochondrial phenotypes in cultured cells. Transgenic mutant embryos derived from this mutant ES cell clone exhibited functionally significant mtDNA depletion in multiple tissues, including brain and muscle, as well as elevations in MMA levels. This model of SCS deficiency and mtDNA depletion will provide a useful tool for exploring the role of a TCA cycle enzyme in the maintenance of mtDNA as well as the molecular pathogenesis of mitochondrial disease with mtDNA depletion.

## RESULTS

### Gene trap screen in mouse ES cells identifies *Sucla2* hypomorphic mutant allele

**Fig. 1.**
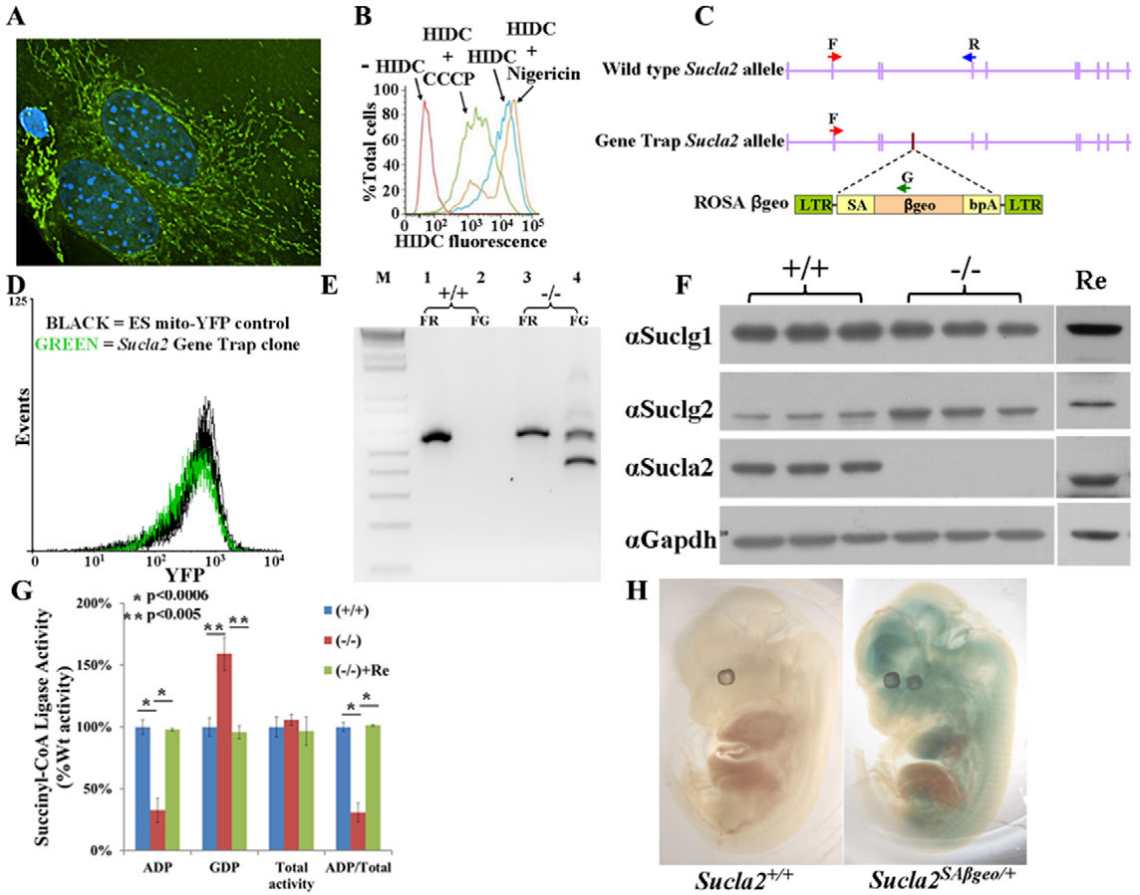
**Gene trap screen for mitochondrial phenotypes identifies *Sucla2* mutant allele.** (A) Wild-type MEFs transfected with mito-YFP and stained with DAPI. (B) Wild-type MEFs stained with DiIC_1_(5) (HIDC) and analyzed by FACS. Cells pre-treated with CCCP (green line), a proton ionophore, exhibit relative depolarization of the MMP. Cells pre-treated with nigericin (orange line), a K^+^-selective ionophore, exhibit relative hyperpolarization of the MMP. (C) Localization of ROSAβgeo gene trap integration into intron 4–5 of *Sucla2* locus. Red (F), blue (R) and green (G) arrows depict relative primer locations for RT-PCR experiments shown in E. (D) FACS analysis of *Sucla2*^+/−^ gene trap ES clone for mito-YFP fluorescence. Each line depicts summary of three independent FACS experiments and shows that the *Sucla2* gene trap clone exhibits an ~30% reduction in mean YFP fluorescence compared with the parental ES cell line. (E) RT-PCR analysis of RNA isolated from *Sucla2* wild-type (+/+; lanes 1–2) and homozygous mutant (−/−; lanes 3–4) MEFs using the primer pairs indicated on the gene map in C. Lanes 1 and 3 represent wild-type allele-specific PCR (FR), generating a 566-bp product. Lanes 2 and 4 represent gene trap allele-specific PCR (FG), generating a 500-bp product from the mutant allele. The smaller second band (380-bp product) in lane 4 is from a gene trap allele-derived transcript from which exon 3 of *Sucla2* splices directly onto βgeo, skipping *Sucla2* exon 4 (sequenced-verified). (F) Western blot analysis of *Sucla2* MEFs. Three independent lines each of *Sucla2*^+/+^ and *Sucla2*^−/−^ MEFs were utilized for western blot analysis of SCS enzyme complex components. Re indicates *Sucla2*^−/−^ MEF cell line rescued by ectopic expression of wild-type *Sucla2* cDNA. (G) Analysis of SCS activities in *Sucla2* MEFs. ADP-specific and GDP-specific SCS activities were measured for wild-type and mutant MEFs. *Sucla2*^−/−^ MEFs exhibit an ADP-specific SCS enzyme deficiency that is rescued by ectopic expression of *Sucla2* (+Re). (H) X-gal staining of wild-type (left) and heterozygous mutant (right) e12.5 embryos shows *Sucla2* expression pattern, predominantly in brain, heart, developing spinal cord and/or neighboring tissues with little expression in liver.

To identify genes important for mitochondrial function that could be candidates for mitochondrial disease genes, a FACS-based genetic screen in mouse ES cells was performed. Two fluorescent markers were chosen as surrogates for mitochondrial mass and mitochondrial membrane potential: first, yellow fluorescent protein (YFP) containing a N-terminal mitochondrial targeting sequence (mito-YFP, Fig. 1A); and, second, 1,1′,3,3,3′,3′-hexamethylindodicarbocyanine iodide [DiIC_1_(5) or HIDC] – a red fluorescent dye that preferentially accumulates in the mitochondrial inner membrane proportional to the mitochondrial inner membrane potential (Fig. 1B) (Mattiasson, 2004). Wild-type mouse ES cells were stably transfected with mito-YFP, transduced with retrovirus packaged with a ROSAβgeo gene trap construct (Fig. 1C) (Friedrich and Soriano, 1991), stained with DiIC_1_(5) and screened for changes in mito-YFP or DiIC_1_(5) fluorescence by FACS. Sorted cells were collected and the stably transduced ES cell clones with gene traps were established by selection in the presence of G418 for neomycin resistance. Established clones were then individually tested for stable differences in mito-YFP fluorescence. Clones that exhibited at least 25% difference in mean mito-YFP fluorescence from the parental cell line were chosen for molecular analysis. Of 379 clones isolated from the screen, 123 clones demonstrated ≥25% difference in mean YFP fluorescence and the gene trap genomic insertion site for 47 of the 123 clones was successfully determined by inverse PCR (supplementary material Table S1). Classes of identified loci included transcriptional regulators (*Trp53*, *Taf5l*, *mft2*), RNA binding proteins (*Pum1*, *Ewsr1*), chromatin modulator (*Smarcad1*), components of signal transduction (*Gpr107*, *Pik3r1*) and metabolic enzymes (*Hk1*, *Sucla2*).

The identification of an ES cell clone with a gene trap of *Sucla2* (Fig. 1C) validates the screening strategy given that *SUCLA2* is a known mitochondrial disease gene that causes mitochondrial encephalopathy with mtDNA depletion (Elpeleg et al., 2005; Carrozzo et al., 2007; Ostergaard et al., 2007b). Because no mouse models for SCS deficiency have been reported, this ES cell clone was chosen for further study and subsequently injected into donor blastocysts to generate a transgenic mouse line. The gene trap construct was inserted in the fourth intron of *Sucla2* (Fig. 1C) and is a hypomorphic allele, with some wild-type transcript detectable by RT-PCR secondary to the typical ‘leakiness’ of gene trapping (Fig. 1E) (Voss et al., 1998).

### *Sucla2* exhibits a tissue-specific expression pattern

The gene trap construct allows for *in situ* detection of expression of the endogenous trapped allele through detection of β-galactosidase activity via X-gal staining (Friedrich and Soriano, 1991). Staining of e12.5 *Sucla2^SAβgeo/+^* embryos demonstrated strong expression of *Sucla2* in the brain, heart, developing spinal cord and/or neighboring tissues with relatively little staining in liver (Fig. 1H). Staining of the e12.5 placenta clearly demonstrated strong expression of *Sucla2* (supplementary material Fig. S1). This expression pattern was consistent with previous reports (Lambeth et al., 2004).

### Mice homozygous for mutant *Sucla2* exhibit late gestational lethality with placental abnormalities

**Fig. 2.**
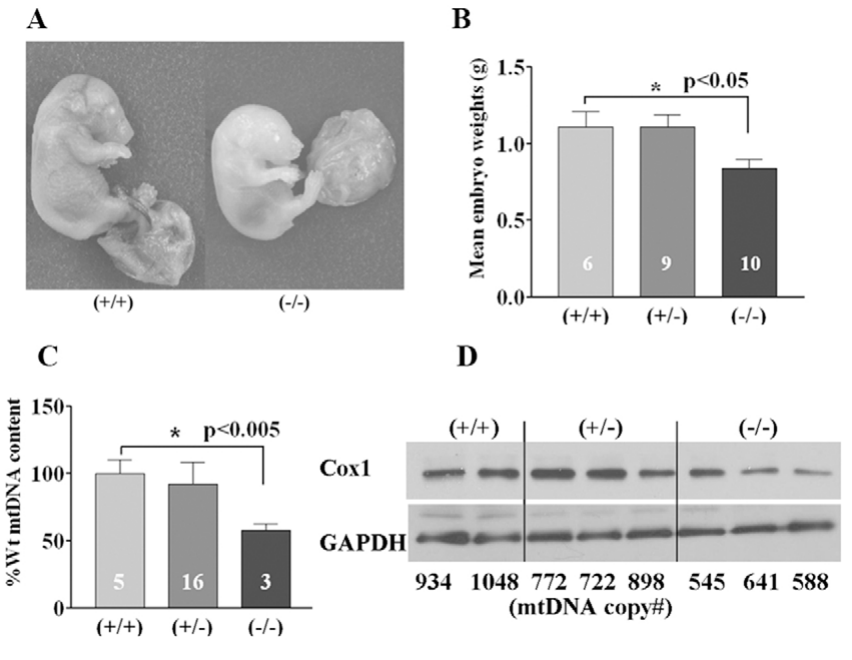
***Sucla2* mutant embryos display growth deficiency and placental mtDNA depletion.** (A) Representative photos of wild-type (left) and homozygous mutant (right) e17.5 embryos. (B) Bar graph depicting average wet weight of e17.5 *Sucla2* embryos (numbers represent sample size for each genotype), with *Sucla2*^−/−^ embryos weighing 25% less. (C) Relative mtDNA content for embryonic placentas from e17.5 *Sucla2* embryos (numbers represent sample size for each genotype). (D) Western blot analysis of e17.5 *Sucla2* placentas. The relative mtDNA copy number for each sample is indicated below each lane of the western blot. COX1 is a mtDNA-encoded subunit of cytochrome *c* oxidase (respiratory chain complex IV).

**Fig. 3.**
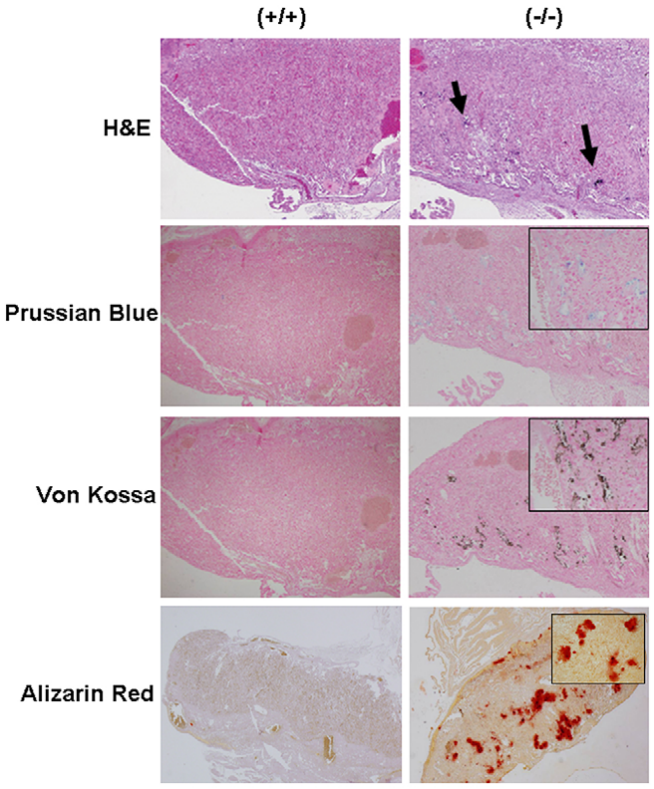
***Sucla2*-deficient placenta exhibit increased mineralization**. Sections of wild-type (left) and mutant (right) placentas from e17.5 *Sucla2* embryos. Sections were stained with H&E (10× magnification), Prussian Blue (for iron) (10× magnification), von Kossa (10× magnification) and Alizarin Red (4× magnification) for calcium. Arrows in upper right panel indicate potential areas of increased mineralization in mutant placenta. Inset boxes show 40× magnification of areas of increased mineralization.

Genetic analysis of progeny from *Sucla2^SAβgeo/+^* heterozygous intercrosses demonstrated that homozygous mutant embryos die late in gestation, predominantly at or after e18.5, with no live born pups identified (Table 1). Western analysis of mouse embryonic fibroblasts (MEFs) established from mutant and wild-type littermate embryos demonstrated a severe reduction of SUCLA2 protein levels associated with a 75% reduction in ADP-specific SCS enzyme activity (Fig. 1F,G; supplementary material Table S2). Interestingly, there was a reciprocal increase in SUCLG2 protein levels that corresponded to a 75% increase in GDP-specific SCS activity that preserved total SCS enzyme activity levels in *Sucla2*^−/−^ MEFs. This increase in SUCLG2 protein levels was mediated at a translational or post-translational level, given that there was no detectable increase in *Suclg2* transcript levels by quantitative real-time PCR (qRT-PCR; supplementary material Table S3). Ectopic expression of wild-type *Sucla2* cDNA in *Sucla2*^−/−^ MEFs restored SCS activities and SUCLG2 protein expression to wild-type levels, demonstrating the specificity of these phenotypes (Fig. 1F,G; supplementary material Table S2). No structural or developmental defects were identified from histopathological analysis of e17.5 mutant embryos (supplementary material Fig. S2); however, the mutant embryos were on average 25% smaller by weight than littermates (Fig. 2A,B) and their placentas exhibited signs of increased mineralization (Fig. 3), suggesting that placental insufficiency might play a pathological role. This was accompanied by a 45% reduction in e17.5 placental mtDNA content by quantitative PCR (qPCR; Fig. 2C; supplementary material Table S4), associated with a trend towards decreased protein levels of COX1, a mtDNA-encoded subunit of cytochrome *c* oxidase (Fig. 2D).

**Table 1.**
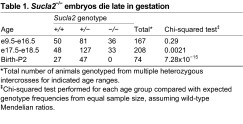
*Sucla2*^−/−^ embryos die late in gestation

* Total number of animals genotyped from multiple heterozygous intercrosses for indicated age ranges.

‡ Chi-squared test performed for each age group compared with expected genotype frequencies from equal sample size, assuming wild-type Mendelian ratios.

### *Sucla2* mutant MEFs demonstrate progressive and functionally significant mtDNA depletion

**Fig. 4.**
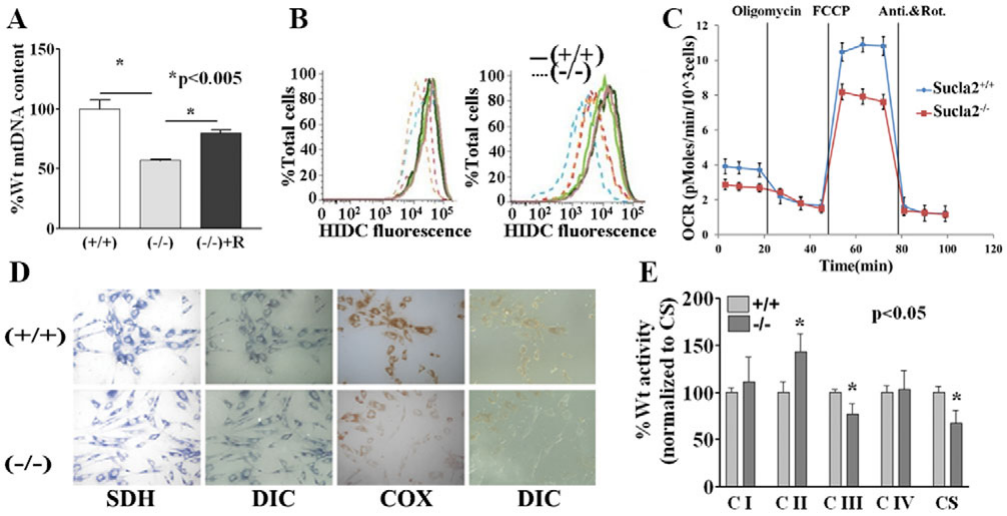
***Sucla2*-deficient MEFs exhibit functionally significant mtDNA depletion associated with relative mitochondrial depolarization, cellular respiration defects and respiratory chain deficiencies.** (A) *Sucla2* mutant MEFs exhibit mtDNA depletion compared with MEFs from wild-type littermates that is rescued by ectopic expression of wild-type *Sucla2* cDNA. (B) The relative mitochondrial membrane potential for *Sucla2* MEFs was determined by staining cells with DiIC_1_(5) (HIDC) followed by FACS analysis (three independent lines for each genotype). The graph on the left shows the analysis soon after the establishment of the MEFs. The graph on the right shows the analysis after 5 weeks of culture with multiple passages. The mutant MEFs demonstrate a progressive relative depolarization of the mitochondrial inner membrane. (C) Cellular respiration analysis of *Sucla2* MEFs demonstrate that *Sucla2*^−/−^ cells exhibit defects in basal respiration, oligomycin-sensitive respiration and respiratory capacity. (D) Histochemical staining of MEFs reveals complex IV deficiency in a subset of *Sucla2*^−/−^ cells. MEFs were stained for SDH (no mtDNA-encoded subunits) and COX (has mtDNA encoded subunits) activities. All cells show uniform staining for SDH, whereas a subset of mutant MEFs exhibit little or no detectable COX activity, in contrast to wild-type cells demonstrating uniform normal staining. (E) Analysis of mitochondrial electron transport chain enzyme activities shows partial deficiency of ETC complex III and a reduction in citrate synthase (CS) activity in *Sucla2* mutant MEFs. CS is a TCA cycle enzyme commonly used as a biochemical marker of mitochondrial matrix content.

Given the histopathological and molecular abnormalities observed in the mutant placentas, *Sucla2* mutant MEFs were also examined for potential mtDNA depletion. MEFs derived from mutant e12.5 embryos and grown in uridine-supplemented media exhibited progressive mtDNA depletion in culture compared with MEFs from wild-type littermates (supplementary material Fig. S3). Mutant MEFs demonstrated a 50% depletion of mtDNA after 5 weeks of culture that was rescued by ectopic expression of wild-type *Sucla2* cDNA (Fig. 4A; supplementary material Table S4). When histochemically stained for succinate dehydrogenase (SDH, no mtDNA-encoded subunits) and cytochrome *c* oxidase (COX, which has three mtDNA-encoded subunits) activities, both wild-type and mutant cells showed uniform staining for SDH. A proportion (~36%) of mutant MEFs exhibited absent or reduced COX activity, in contrast to wild-type cells demonstrating uniform COX staining (Fig. 4D; supplementary material Fig. S4). Measurement of enzyme activities of individual electron transport chain (ETC) complexes from cell lysates (normalized to citrate synthase activity) showed a significant reduction in complex III and citrate synthase activities, whereas complex II (SDH) activity was mildly increased (Fig. 4E; supplementary material Table S5). The cells were further analyzed by FACS-based analysis of relative mitochondrial membrane potential using the potential-sensitive dye DiIC_1_(5) (Fig. 1B). After 5 weeks of culture, mutant MEFs demonstrated a relative partial depolarization of the mitochondrial inner membrane (Fig. 4B) in parallel with the mtDNA depletion and ETC deficiencies described above (Fig. 4A,D,E). Furthermore, the cellular respiration of MEFs was analyzed by measuring oxygen consumption when grown in the presence of pyruvate and glucose and sequentially exposed to ETC inhibitors and uncouplers. Oligomycin (an ATPase and complex V inhibitor), carbonylcyanide-*p*-trifluoromethoxyphenylhydrazone (FCCP, a mitochondrial uncoupler) and rotenone plus antimycin A (inhibitors of complex I and complex III, respectively) were used in order to measure basal respiration, oligomycin-sensitive respiration (typically reflecting complex V activity) and total respiratory capacity, respectively (Fig. 4C). This analysis demonstrated that the mutant MEFs show significant defects in basal respiration, oligomycin-sensitive respiration and total respiratory capacity (Fig. 4C; supplementary material Fig. S5). In combination, these studies suggest that progressive mtDNA depletion in *Sucla2* mutant cells ultimately interferes with proper steady-state expression of mtDNA-encoded subunits, resulting in deficiency of respiratory chain complexes containing mtDNA-encoded subunits, mitochondrial depolarization and perturbation of mitochondrial respiration.

### *Sucla2* mutant embryos exhibit progressive and functionally significant mtDNA depletion with elevated levels of MMA

**Fig. 5.**
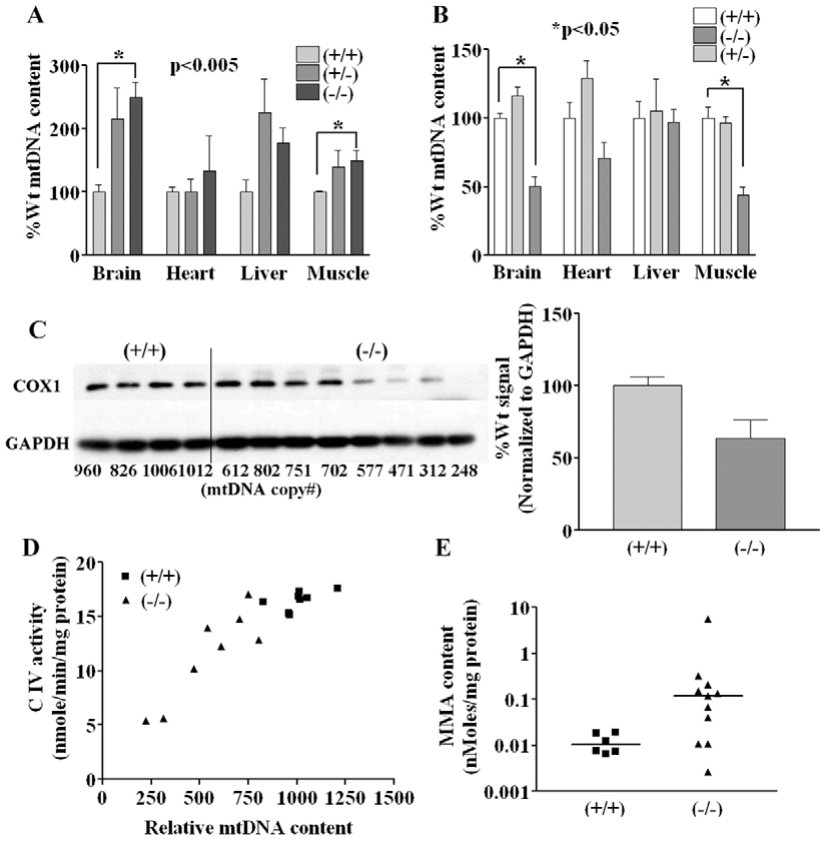
***Sucla2*-deficient embryo tissues exhibit progressive, functionally significant mtDNA depletion and elevated MMA.** (A) Relative mtDNA content of tissues from e15.5 *Sucla2* tissues (*n*=3 for each genotype). (B) Relative mtDNA content of tissues from e17.5 *Sucla2* tissues (*n*=8 for each genotype) showing progressive, significant mtDNA depletion in brain and skeletal muscle. (C) Western blot analysis of e17.5 *Sucla2* brain tissues. The relative mtDNA copy number for each sample is indicated below each lane of the western blot. Severe reduction in COX1 expression is observed when the relative mtDNA copy number falls below 600. The bar graph shows quantification of the band intensities of the blot shown. (D) Complex IV deficiency is proportional to mtDNA content in *Sucla2*^−/−^ brain. Brains from eight wild-type and eight mutant e17.5 embryos were used for analysis of mitochondrial complex IV activity and for relative mtDNA content. Graph depicts relationship of complex IV activity and mtDNA content. Mutant brains with relative mtDNA copy number below ~600 exhibit proportional loss of complex IV activity. (E) *Sucla2*^−/−^ brains exhibit increased levels of MMA. Brain lysates from wild-type and mutant e17.5 embryos were used for measurement of MMA levels normalized to total cellular protein content.

To examine the potential effect of *Sucla2* deficiency in other tissues, analysis of mtDNA content in various embryonic tissues was performed. For e15.5 embryos, no mtDNA depletion was detectable in brain, heart, skeletal muscle or liver (Fig. 5A; supplementary material Table S4). In fact, there was a significant increase in mtDNA content in homozygous mutant brain and muscle compared with wild-type littermates. Interestingly, this phenomenon was also detected during the first week of culture of *Sucla2* heterozygous and homozygous mutant MEFs grown under various conditions (supplementary material Fig. S3). However, by embryonic stage e17.5, *Sucla2* mutant animals demonstrated significant mtDNA depletion (50–55%) in brain and skeletal muscle, a trend towards mtDNA depletion in heart and no depletion in liver (Fig. 5B; supplementary material Table S4). Western blot analysis of COXI in brain extracts showed that *Sucla2* mutants exhibit reduced COXI levels proportional to the relative amount of mtDNA depletion (Fig. 5C), consistent with the interpretation that severe mtDNA depletion in cells prevents proper expression of mtDNA-encoded components, resulting in functional deficits. This interpretation is also supported by the observation that the degree of relative enzyme deficiency for ETC complexes containing mtDNA-encoded subunits was proportional to the degree of mtDNA depletion in mutant brains (Fig. 5D; supplementary material Fig. S6). Additionally, examination of the cellular content of MMA in brain extracts demonstrated increased levels in the majority of mutants (Fig. 5E), reminiscent of increased serum MMA observed in patients, presumably secondary to increased succinyl-CoA resulting from SCS deficiency (Ostergaard et al., 2007b).

## DISCUSSION

### *Sucla2* mutant mice recapitulate the molecular and biochemical features of *SUCLA2*-dependent mtDNA depletion syndrome

In this study, a genetic screen designed to identify genes that, when mutated, confer abnormal mitochondrial phenotypes in cells resulted in the isolation of gene trap allele of *Sucla2*, the mouse ortholog of the ADP-specific β-isoform of SCS. Mouse embryos mutant for *Sucla2* exhibit deficiency of ADP-specific succinyl-CoA synthetase activity, significant depletion of mtDNA in brain and skeletal muscle and increased cellular content of MMA, which are prominent features observed in patients that have mitochondrial encephalomyopathy with mtDNA depletion associated with *SUCLA2* mutations (Elpeleg et al., 2005; Carrozzo et al., 2007; Ostergaard et al., 2007b). *Sucla2* mutant MEFs demonstrate functionally significant mtDNA depletion associated with reduced levels of mtDNA-encoded proteins, leading to respiratory chain deficiencies, partial depolarization of the mitochondrial inner membrane and cellular respiration defects. An obvious difference between *Sucla2*-deficient mice and *SUCLA2* patients is that *Sucla2*^−/−^ mice experience late gestational embryonic lethality, whereas human patients (including patients with homozygous frameshift mutations) appear normal at birth following an uneventful pregnancy, develop symptoms during infancy and typically succumb to their disease during childhood. In contrast, the *Sucla2* mutant embryos are smaller than wild-type littermates and their placentas exhibit increased mineralization and mtDNA depletion, which might cause placental insufficiency or dysfunction contributing to embryonic lethality. In humans, mineralization (or calcification) of placenta at term gestation (i.e. after 36 weeks gestation) is considered normal, as placental calcium content increases with gestational age (Poggi et al., 2001). However, significant placental calcification prior to 36 weeks is typically not normal and has been linked to pregnancy-induced hypertension and fetal growth restriction (Chen et al., 2012). In addition, villous trophoblastic basement membrane (TBM) calcifications are associated with congenital disorders and fetal thrombotic vasculopathy (Chen et al., 2012). Increased preterm placental calcification is considered to be a predictor of poor uteroplacental blood flow and adverse pregnancy outcomes (Chen et al., 2012). Increased placental calcification could reflect abnormalities in fetal calcium utilization and excretion and/or impaired calcium metabolism resulting in hypoxic stress in preeclamptic syncytiotrophoblasts (Yang et al., 2013). *Sucla2* mutant placenta shows coarse calcifications that appear to be within spongiotrophoblasts (Fig. 3), rather than the maternal blood space, as seen in the case of human preterm placental calcifications. Because mitochondrial function and energy metabolism are important for cellular calcium handling (Nunnari and Suomalainen, 2012), it is possible that *Sucla2* deficiency and energy metabolism dysfunction alters murine placental calcium handling and metabolism, resulting in placental calcification. Currently, it is unclear whether the placental calcifications are cell autonomous consequences of *Sucla2* deficiency in the placenta and/or the result of decreased uteroplacental blood flow.

Although human and murine placentas are similar in many aspects, there are distinct anatomical and cellular differences that might make murine placentas more susceptible to SCS deficiency (Malassiné et al., 2003). Human and mouse placenta differ from each other in terms of morphogenesis. A definitive structure of placenta is observed as early as day 21 of pregnancy in humans whereas in mouse a definitive structure is not apparent until midway through gestation (Malassiné et al., 2003). This shortened period of placental maturity relative to the gestational period could render the mouse placenta particularly susceptible to stress induced by defective utilization of nutrients and/or impaired calcium metabolism in the context of mitochondrial dysfunction. In addition, the giant trophoblastic cells of mouse are not analogous to their human counterparts. Mouse giant cells are generated by endoreplication (Soares et al., 1996), which is not the case in humans. The direct nutrient uptake of fetal nutrition from circulating maternal blood by trophoblast cells also differs between mouse and human placentas. The murine labyrinthine structure allows countercurrent exchanges between maternal and fetal capillaries arranged in parallel to each other. In humans, the multivillous structure results in an intermediate between a countercurrent and a parallel-flow system (Leiser and Kaufmann, 1994). Whether the differences in placental morphogenesis and/or maternofetal nutrient exchange between mouse and human contributes to the placental mineralization observed in *Sucla2* mutant placenta is unknown and requires further investigation. It is also important to note that mice deficient for methylmalonyl-CoA mutase exhibit extremely high levels of MMA, but are indistinguishable from wild-type littermates at birth, and subsequently die within 24 hours of life (Peters et al., 2003). Therefore, it is unlikely that toxicity from elevated levels of MMA per se significantly contributes to the late gestational lethality of *Sucla2* mutant embryos.

### What is the mechanism for mtDNA depletion in Sucla2 deficiency?

How deficiency of *Sucla2* (SUCLA2) leads to mtDNA depletion is currently not well understood. Previous studies have suggested that SCS forms a complex with a mitochondrial isoform of nucleotide diphosphate kinase (NDPK) (Kavanaugh-Black et al., 1994; Kowluru et al., 2002). In addition, knockdown of *SUCLG2* in *SUCLA2*-deficient fibroblasts reportedly results in mtDNA depletion and reduction in NDPK activity (Miller et al., 2011), suggesting that disruption of a SCS-NDPK complex leads to a perturbation of mitochondrial nucleotide (dNTP) pools that affects mtDNA replication. Perturbation of mitochondrial nucleotide pools associated with mtDNA depletion has been demonstrated in cellular and animal models of thymidine phosphorylase deficiency (López et al., 2009; González-Vioque et al., 2011). Deficiency of *Sucla2* results in a severe reduction in ADP-specific SCS activity and a reciprocal increase in GDP-specific SCS activity in MEFs (Fig. 1G). Because substrate-level phosphorylation of GDP by SCS is the only source of metabolically generated GTP in the mitochondrial matrix, changes in GDP-specific SCS activity could result in perturbations of mitochondrial GTP content. Therefore, altered ADP- and GDP-specific activities could directly affect mtDNA replication or might have broader regulatory effects, much like those demonstrated with glucose-stimulated insulin secretion in an insulinoma cell line and isolated rat islet cells (Kibbey et al., 2007). Alternatively, SUCLA2 might be a component of the mtDNA nucleoid; loss of nucleoid components can lead to missegregation of mtDNA and loss of mtDNA copy number, as has been demonstrated in yeast lacking another TCA cycle enzyme, aconitase (Chen et al., 2005). Further studies will be required to address the potential mechanisms of mtDNA depletion with *Sucla2* deficiency.

### Gene trap mutagenesis and FACS in ES cells is an effective strategy for identifying genes important for mitochondrial function

The genetic screen described in this report, utilizing gene trap mutagenesis and FACS for surrogate mitochondrial fluorescence markers, was designed to identify genes that when mutated cause abnormal mitochondrial phenotypes. The isolation of a mouse ES cell clone with a mutation in *Sucla2*, a known mitochondrial disease gene, validates the utility of this approach. It is important to note that the isolated gene trap ES cell clones are at most haploinsufficient for mutated loci, whereas mutations in known nuclear-encoded mitochondrial disease genes are typically recessive (Graham, 2012). This suggests that the surrogate fluorescence markers for mitochondrial mass and mitochondrial membrane potential can detect subtle phenotypes in cells with heterozygous mutations in genes that cause recessive phenotypes, as described here for *Sucla2* (Fig. 1D). In fact, the presence of subtle phenotypes in heterozygous mutant *Sucla2* cells is also suggested by the detection of increased relative mtDNA content (compared with wild type) in early passage *Sucla2*^−/−^ MEFs (supplementary material Fig. S3) and e15.5 *Sucla2*^−/−^ brain and muscle (Fig. 5A; supplementary material Table S4). This phenomenon could reflect a compensatory response to loss of SCS activity and/or possible perturbation of mitochondrial nucleotide pools that ultimately fails, resulting in progressive mtDNA depletion.

This genetic screen identified over 20 genes that are involved in a wide array of cellular processes, including transcriptional regulation, post-transcriptional regulation, chromatin modulation, signal transduction and metabolism (supplementary material Table S1). In 2010, Yoon et al. described a RNAi screen in cultured C2C12 cells that utilized a similar surrogate fluorescence mitochondrial marker strategy from which over 150 genes involved in a comparably diverse array of biological processes were identified (Yoon et al., 2010). Interestingly, only *Smarcad1* was identified in both screens, which could be due to inherent differences in the screen designs, including cell type (ES cell versus C2C12 muscle cell), form of mutagenesis (gene trap versus RNAi), inherent non-saturating nature of the screens and thresholds for the reproducible change in marker fluorescence. A distinct advantage of performing a genetic screen in mouse ES cells is that transgenic animals can be generated for organismal studies using mutant ES cells, as described in this report. In summary, screening for genes important for mitochondrial function by utilizing gene trap mutagenesis and FACS in mouse ES cells is an effective approach that offers the potential to generate novel animal models and to identify genes that might not be identified from screens in other cell types.

## MATERIALS AND METHODS

### Gene trap mutagenesis of mouse ES cells

Mouse ES cells were maintained and grown on mitomycin C-inactivated MEFs (STO cells) in the presence of LIF using standard ES cell techniques (Conner, 2001). AB2.2 129SvEv mouse ES cells were stably transfected with a mitochondrial-targeted EYFP construct (pMito-EYFP, Clontech) that was modified by exchanging the Neo^R^ cassette for Puro^R^ cassette using standard PCR cloning techniques. Individual Puro^R^ ES cell clones were individually analyzed by fluorescence microscopy and FACS for YFP fluorescence. The clones that reproducibly demonstrated high, uniform mitochondrial fluorescence were chosen for gene trapping experiments. The ROSAbgeo gene trap construct was transfected into the GP+E86 packaging cell line to derive a retroviral producer cell line; virus titers were determined; and ES cell infections were performed as previously described (Friedrich and Soriano, 1991). ES cell infections were performed using a multiplicities of infection (MOI) of 0.1 to reduce the possibility of multiple integrations per cell.

### Cell sorting of transduced ES cells

Twenty-four hours after retroviral infection and prior to drug selection, ES cells were stained with 100 nM DiIC_1_(5) (Invitrogen) for 30 minutes at 37°C, 5% CO_2_. In parallel, wild-type cells were treated with 50 μM CCCP (Sigma) and 100 nM nigericin (Sigma) at 37°C, 5% CO_2_, for 30 minutes as controls for depolarized and hyperpolarized mitochondria, respectively. After incubation, cells were washed once with warm phosphate-buffered saline (PBS) and then harvested and pelleted by centrifugation. Cells were then analyzed and sorted using a FACSAria sorter utilizing blue (488 nm) and red (638 nm) lasers for excitation and appropriate emission filters to detect YFP and DiIC_1_(5) (similar to Cy5) fluorescence. With the goal of screening approximately 10^5^ gene trap events, 10^7^ cells exposed to virus were harvested for sorting, assuming that approximately 10% of infected cells will have true gene traps. Two batches of transduced ES cells were sorted by FACS separately; one for changes in YFP fluorescence and one for changes in DiIC_1_(5) fluorescence. Cells that exhibited the 0.1% highest and 0.5% lowest green (YFP fluorescence) or red DiIC_1_(5) fluorescence were collected, plated on STO feeder cells and then placed under G418 selection (100 mg/ml) the next day. After 10–14 days of selection, 379 individual clones were picked [98 clones screened for changes in YFP fluorescence and 281 clones screened for changes in DiIC_1_(5) fluorescence], established and subsequently analyzed for stable changes in YFP fluorescence by FACS analysis. The vast majority of identified clones (45/47) demonstrated a stable increase in YFP fluorescence, suggesting an increase in mitochondrial mass.

### Inverse PCR to identify gene trap genomic insertion sites

To identify the genomic insertion site of gene trap ES cell clones, inverse PCR was performed. ES cell clones were grown in individual wells in a 96-well plate format and genomic DNA was isolated in the plate as previously described (Graham et al, 1997). Approximately 1 μg of genomic DNA was subsequently digested with 10 units of *Hin*dIII, *Fsp*I or *Pvu*I, and then purified over a Qiagen QIAquick spin column. The recovered DNA was then subjected to intramolecular ligation by adding 10 units of T4 ligase in 50 μl total volume. After the ligation, the DNA was precipitated in the presence of 300 mM sodium acetate and ethanol, washed in 70% ethanol and the pellet resuspended in TE buffier. The isolated ligated DNA was then subjected to nested PCR reactions using the following ROSAβgeo-specific primers: for the first round of PCR, PreU5SP (5′-ACCAATCAGTTCGCTTCTCG-3′) and PreSASPrev (5′-CCAGGGTTTCCTTGATGATG-3′); for the second round of PCR, U5SP (5′-GAGACCCTCCCAAGGAACAG-3′) and SASPrev (5′-CAAACTCTTCGCGGTCTTTC-3′). DNA fragments amplified by nested PCR were then analyzed by agarose gel electrophoresis, purified and sequenced to determine genomic DNA insertion site (supplementary material Table S1).

### Generation of transgenic animals

To generate transgenic animals, cells from the *Sucla2^SAβgeo/+^* ES cell clone (derived from AB2.2 129SvEv ES cell line) were microinjected into C57BL/6 blastocysts to generate chimeras. Procedures were carried out by the Baylor College of Medicine Genetically Engineered Mouse Core Laboratory using standard protocols. Germline-transmitting male chimeras were bred with C57BL/6 females to establish the mouse line and all studies therefore were performed using mice on a 129SvEv/C57BL/6 mixed genetic background. All animal experiments performed conformed to protocols approved by the Baylor College of Medicine IACUC.

### Genotyping and RT-PCR

*Sucla2* mice were genotyped by multiplex PCR using a common forward primer (Sucla2F), and allele-specific reverse primers for wild-type (Sucla2R), and gene trap (ROSABgeoR) alleles (all primer sequences are available on request). PCR products were subjected to agarose gel electrophoresis and the wild-type and mutant bands were identified on the basis of size (wild-type, 963 bp; mutant, 1073 bp). Reverse transcription of 1 μg of RNA was performed using the iScript cDNA synthesis kit (Bio-Rad). A common forward *Sucla2* exon primer (Sucla2E2F or ‘F’ in Fig. 1C) and allele-specific reverse primers for wild-type (Sucla2E5R or ‘R’ in Fig. 1C) and gene trap (BgeoR or ‘G’ in Fig. 1C) alleles were used for allele-specific PCR reactions to generate 566-bp wild-type and 500-bp gene trap PCR products, respectively.

### Histology

Embryos and placentas were fixed in 10% neutral buffered formalin prior to weighing and dissection. No gross external or internal malformations were identified. Tissue samples from major organs and the placenta were routinely processed and paraffin embedded. Paraffin sections (3 micron) were cut and stained with hematoxylin and eosin (H&E). Duplicated 3-micron sections were stained for iron (Prussian Blue reaction) and calcium (von Kossa’s silver nitrate reaction). A separate 3-micron section was stained for calcium with Alizarin Red. Stained tissue sections were pictured using Nikon Eclipse 90i microscope and NIS-Elements software from Nikon.

### Western blotting

Whole cell lysates from tissues or MEFs were prepared in standard RIPA buffer. After centrifugation of the lysate, soluble proteins were isolated in the supernatant and protein concentration determined according to the Bradford-Lowry method. Protein samples were separated on a 10% SDS-polyacrylamide gradient mini-gel. Proteins were transferred electrophoretically to 0.45-mm polyvinylidine difluoride membrane for 75 minutes at 100 V. Membranes were blocked for 3 hours in 5% milk-PBS and incubated overnight with antibodies. After three washes with PBS containing 0.05% Tween 20, the membranes were incubated for 2 hours with horseradish-peroxidase-conjugated goat anti-rabbit or goat anti-mouse (Bio-Rad) diluted in 5% milk-PBS. The secondary antibody was detected using the chemiluminescent ECL Plus reagent (Millipore). Band intensities from autoradiographs were quantified using NIH ImageJ software. Primary polyclonal antibodies used were rabbit α-Sucla2 (1:200; Santa Cruz Biotechnology), rabbit α-Suclg1 (1:10,000; Gene Tex) and rabbit α-Suclg2 (1:10,000; Gene Tex). Primary mouse monoclonal antibodies used were α-COXI (1:1000; MitoSciences) and α-GAPDH (1:100,000; Gene Tex) as loading control.

### X-gal staining

e12.5 embryos from a cross of *Sucla2*^+/+^ and *Sucla2*^SAβgeo/+^ mice were isolated and washed with PBS containing 2 mM MgCl_2_ to remove any traces of blood. Embryos and placenta were then fixed in 4% paraformaldehyde for 2 hours and washed with PBS containing 2 mM MgCl_2_. Embryos were incubated with X-gal reaction buffer (5 mM potassium ferrocyanide, 5 mM potassium ferricyanide, 2 mM MgCl_2_, 0.02% Nonidet P-40, 0.01% Na deoxycholate and 1 mg/ml X-gal) overnight at 37°C, washed with PBS containing 2 mM MgCl_2_ and dehydrated. Embryos were briefly incubated in methyl salicylate (Sigma) to clear the tissue and then photographed.

### Cell culture

MEFs were generated from e12.5 embryos. The embryonic sac was separated from fetal material and cell suspensions were generated from a small portion of embryo (avoiding organs, head and limbs) using 10 mg/ml collagenase H. A portion of whole-cell suspension was taken for genotyping and the rest was plated in a 96-well plate in embryo fibroblast culture medium (cell culture media compositions are available on request). For genetic rescue of *Sucla2*^−/−^ MEF cell line, the full-length *Sucla2* cDNA was subcloned into pINDUCER (Meerbrey et al., 2011) that was modified by exchanging the Neo^R^ cassette for Puro^R^. The *Sucla2*-pINDUCER(Puro^R^) construct was stably transfected into *Sucla2*^−/−^ MEFs by electroporation. Ectopic expression of *Sucla2* was induced by exposing cells to 100 ng/ml doxycycline for a minimum of 72 hours.

### Mitochondrial membrane potential measurement

Cells used for mitochondrial membrane potential (MMP) measurement were plated in a six-well plate and DiIC_1_(5) (Invitrogen) was added to a final concentration of 50 nM. The cells were then incubated at 37°C, 5% CO_2_, for 30 minutes. In parallel, wild-type cells were treated with 50 μM CCCP (Sigma) and 100 nM nigericin (Sigma) at 37°C, 5% CO_2_, for 30 minutes as controls for depolarized and hyperpolarized mitochondria, respectively. Cells were harvested and then analyzed on a LSRII flow cytometer with 633 nm excitation using emission filters appropriate for Alexa-Fluor-633 dye. Flow Jo software was used to analyze the data. At the concentration of nigericin used, a small proportion of the wild-type cells exhibited uncoupling (Fig. 1B), probably due to drug toxicity.

### qPCR and qRT-PCR

Relative mtDNA content of the MEFs and various tissues were analyzed using real-time qPCR, as described before (Bai and Wong, 2004) with the following modifications. The β2 microglobulin gene (*B2M*) was used as the nuclear gene (nDNA) normalizer for calculation of the mtDNA/nDNA ratio. The ND1 region of mouse mtDNA was amplified using forward primer, ND1F, and reverse primer, ND1R, giving an amplicon of 160 bp. A fragment of *B2M* gene was amplified using forward primer, B2MF, and reverse primer, B2MR, giving an amplicon of 106 bp. The relative mtDNA content (mtDNA/B2M ratio) was calculated using the formula: mtDNA content = 1/2^ΔCt^, where ΔC_t_ = C_t_^mtDNA^ − C_t_^B2M^. RNA was isolated from three different wild-type and Sucla2 mutant cell lines. Reverse transcription of 1μg of RNA was performed using the iScript cDNA synthesis kit (Bio-Rad) and the cDNA was used to perform qRT-PCR. The fold change of SCS components was measured using the ΔΔCt method.

### Cellular respiration assay

The XF24 extracellular flux analyzer (Seahorse Biosciences) was used to measure the rates of MEF oxygen consumption. Cells were plated the day prior to the experiment on XF24 cell culture 24-well microplates at a density of 60,000 cells per well. XF assay media (5 mM glucose, 2 mM pyruvate in unbuffered DMEM; Seahorse Biosciences) was prepared and the pH adjusted to 7.0 on the day of the experiment. XF assay media was used to prepare cellular stress reagents: 500 nM oligomycin, 500 nM FCCP, 100 nM antimycin A and 100 nM rotenone (final concentrations). All the reagents were loaded into the injection ports as recommended by Seahorse Biosciences. Oxygen consumption rates (OCR) were cyclically measured with each of the 12 cycles consisting of 3 minutes mixing, 2 minutes equilibration and 3 minutes OCR measurements. After the assay was completed, viable cells in each well were counted using a Vi-Cell XR cell counter and the cell counts used to normalize the OCR rates, with OCR being expressed as pmoles oxygen/minute/10^3^ cells.

### ETC and SCS enzyme assays

Enzymatic assays of respiratory electron transport chain (ETC) complexes I-IV and citrate synthase were performed as described before (Graham et al., 2010) using a minimum of 25 mg of tissue or 10^7^ cells. Briefly, complex I activity (NADH:ubiquinone oxidoreductase) was determined by measuring oxidation of NADH at 340 nm (using ferricyanide as the electron acceptor). Complex II activity (succinate dehydrogenase) was determined by measuring the reduction of the artificial electron acceptor 2,6-dichlorophenol-indophenol (DCIP) at 600 nm. Complex III activity (ubiquinol:cytochrome *c* oxidoreductase) was determined by measuring the reduction of cytochrome *c* at 550 nm. Complex IV activity (cytochrome *c* oxidase) was determined by measuring the oxidation of cytochrome *c* at 550 nm. Citrate synthase activity was determined by measuring the reduction of 5,5′-dithiobis(2-nitrobenzoic acid) (DTNB) at 412 nm, which is coupled to the reduction of acetyl-CoA by citrate synthase in the presence of oxaloacetate. Details of the reaction mixtures are available on request.

Succinyl-CoA synthetase (SCS) activity was measured at 30°C in whole cell lysates from tissues or MEFs in the direction of the succinate to succinyl-CoA reaction, as previously described (Lambeth et al., 2004) with some modifications. The complete assay mixture in a volume of 175 μl contained 50 mM potassium phosphate, pH 7.2, 10 mM MgCl_2_, 0.2 mM succinyl-CoA, 2 mM ADP (for A-SCS) or 1 mM GDP (for G-SCS), and 0.2 mM DTNB. The reactions were initiated by adding succinyl-CoA and DTNB in quick succession to the above mixture along with cell lysates containing 5 μg of protein. Rates were corrected by subtracting the rate observed in the absence of ADP or GDP. The release of CoA-SH from succinyl-CoA was measured spectrophotometrically at 412 nm, indicating the formation of thionitrobenzoate from the interaction of CoA-SH with DTNB. All activities were calculated as nmoles/minute/milligram protein and expressed as a percentage of control activity.

### Histochemical staining of COX and SDH activities

Cells to be stained for COX or SDH activity were grown on sterile glass coverslips overnight. Growth medium was removed from the cells and the culture dish placed onto a bed of ice-water slurry. The cells then rinsed three times with ice-cold PBS and once with double-distilled H_2_O, and all traces of fluid removed. Cells were air-dried for 3 minutes and then incubated at 37°C with freshly prepared staining buffer. Histochemical staining was performed as previously described (De Paepe et al., 2009) with some modifications. For COX, the staining buffer consisted of 10% sucrose, 100 μM of fully reduced bovine cytochrome C, 8 units catalase, 1 mg/ml 3,3′-diaminobenzidine (DAB) and 0.25% DMSO in 20 mM sodium phosphate buffer (pH 7). Cells to be stained for SDH activity were incubated in 0.1 M phosphate buffer (pH 7) containing 1.5 mM nitroblue tetrazolium, 130 mM sodium succinate, 0.2 mM phenazine methosulfate and 1.0 mM sodium azide for 1–2 hours at 37°C.

After staining, the cells were rinsed once with 20 mM phosphate buffer, and once with ice-cold methanol, then re-hydrated and mounted in Vectashield. Cells were imaged using a Nikon Eclipse 90i microscope and NIS-Elements software from Nikon. Each cover slip was divided into quadrants and the number of cells (total and positive for staining) were counted manually. Cells positive for staining were expressed as percentage of total cells. Standard deviation was calculated by Student’s *t*-test.

### Measurement of MMA

Tissue content of MMA was determined by liquid chromatography combined with tandem mass spectrometry (HPLC-MS/MS) using a method modified from previous publications (Kushnir et al., 2001; Schmedes and Brandslund, 2006). Briefly, 500 μl of tissue extract was mixed with 90 μl of concentrated phosphoric acid and then extracted with 3 ml of *tert*-butylmethyl ether (MTBE). Next, the dried supernatant was derivatized with butanolic HCl at 65°C for 20 minutes. The analysis was performed on a Waters 2695 Alliance HPLC system connected to a Micromass Quattro Micro tandem mass spectrometer. The column (Waters Symmetry C8, 3.5 μm, 2.1×100 mm) was eluted isocratically with 85% methanol containing 5 mM ammonium formate. Positive ion mode was used. The multiple-reaction monitoring selected for MMA and d3-MMA were m/z 231>119 and 234>122, respectively. Nebuliser gas flow was set to 20 l/hour and desolvation gas 580 l/hour. Cone voltage was 35 V, capillary 3800 V and collision 10 V. The amount of MMA was normalized to the protein content of the tissue extract.

## Supplementary Material

Supplementary Material
