# Supplementary material for: Screen for abnormal mitochondrial phenotypes in mouse embryonic stem cells identifies a model for succinyl-CoA ligase deficiency and mtDNA depletion
Source: Dis Model Mech. 2013 Nov 21;7(2):271–80. doi: 10.1242/dmm.013466 (PMC3917248; doi:10.1242/dmm.013466)
Supplement: Supplementary Material [file supp_7_2_271__index.html]

Screen for abnormal mitochondrial phenotypes in mouse embryonic stem cells identifies a model for succinyl-CoA ligase deficiency and mtDNA depletion — Supplementary Material 

# Screen for abnormal mitochondrial phenotypes in mouse embryonic stem cells identifies a model for succinyl-CoA ligase deficiency and mtDNA depletion

## DMM013466 Supplementary Material

**Files in this Data Supplement:**

- **Supplementary Material PDF**
