# Supplementary material for: Screen for abnormal mitochondrial phenotypes in mouse embryonic stem cells identifies a model for succinyl-CoA ligase deficiency and mtDNA depletion
Source: Dis Model Mech. 2013 Nov 21;7(2):271–80. doi: 10.1242/dmm.013466 (PMC3917248; doi:10.1242/dmm.013466)
Supplement: Supplementary Material [file supp_7.2.271_DMM013466.pdf]

Figure S1

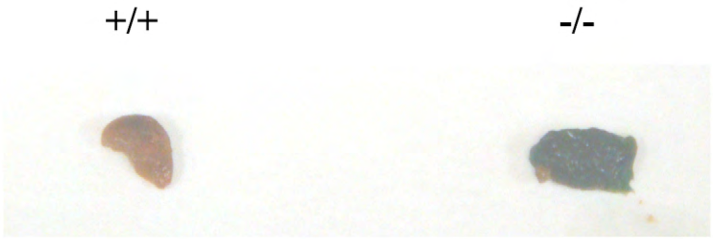

Fig. S1. X-gal staining of wild-type and heterozygous mutant e12.5 placenta. X-gal staining of e12.5 placenta (left, wild type; right, heterozygous mutant) taking advantage of β-galactosidase activity of gene trap construct demonstrates strong expression of *Sucla2* in the placenta.

Figure S2

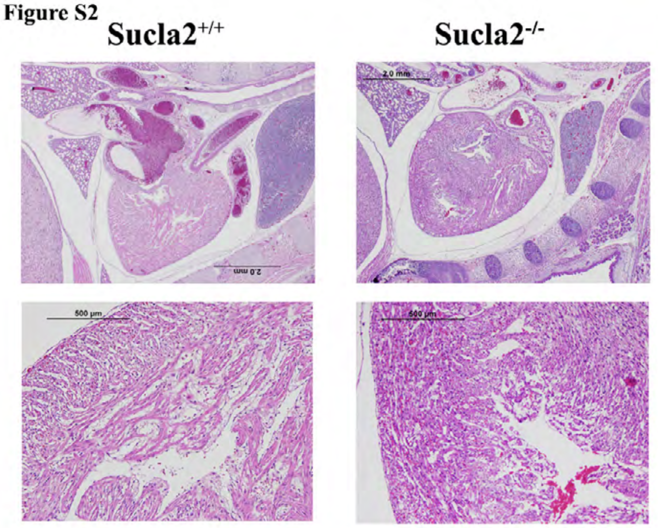

Fig. S2. *Sucla2*$^{-/-}$ mutant embryos exhibit normal cardiac morphology. Panels show H&E stained sections of *Sucla2* wild type (left) and mutant (right) hearts dissected from e17.5 embryos. No structural or morphological abnormalities were detected in mutant hearts or any other tissues.

Figure S3

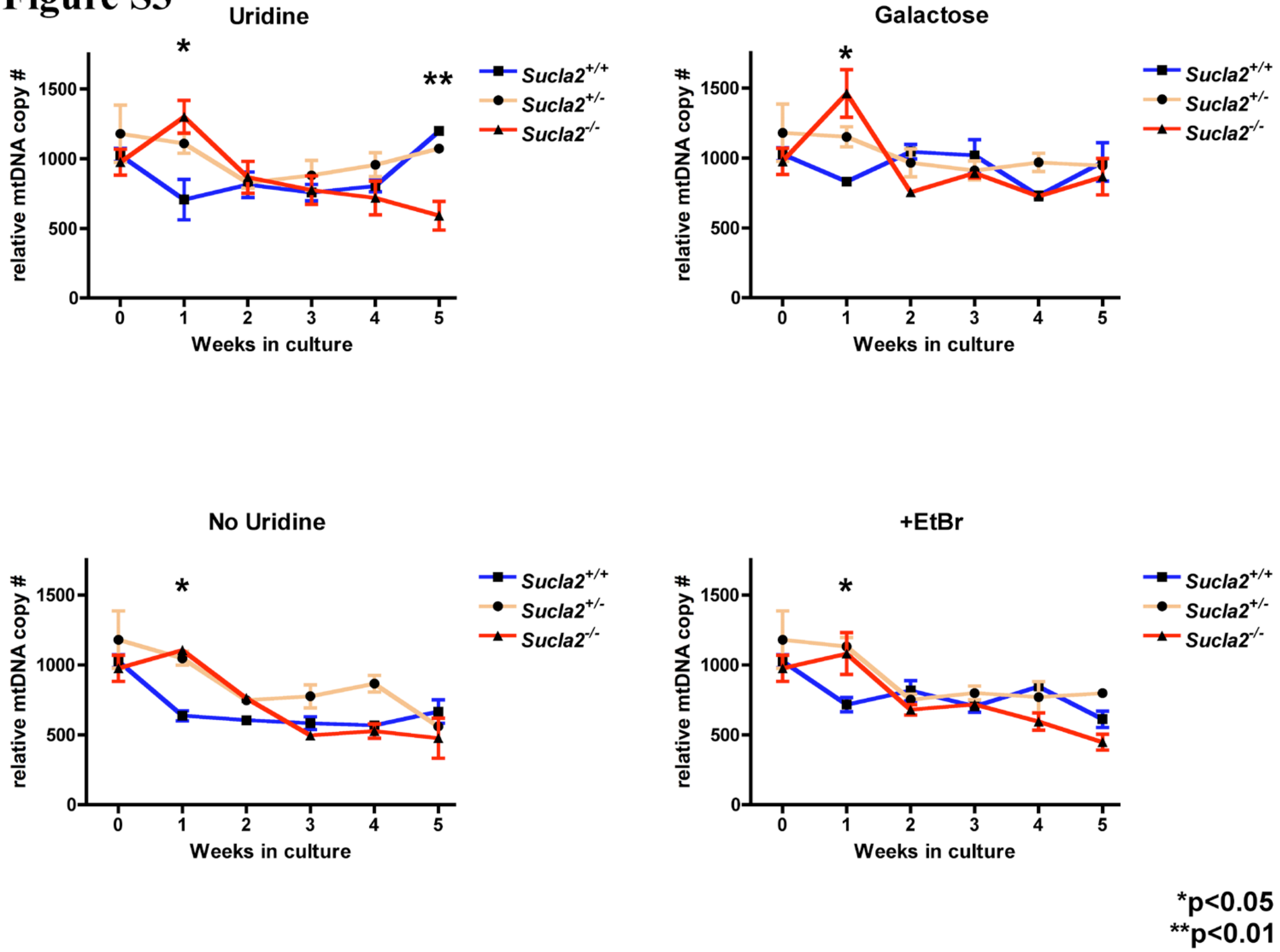

Fig. S3. mtDNA content of *Sucla2* mutant MEFs dependent on growth conditions. Graphs depict relative mtDNA content of *Sucla2*$^{+/+}$, *Sucla2*$^{+/-}$, and *Sucla2*$^{-/-}$ MEFs (three independent cell lines for each genotype) at weekly intervals over a 5-week period under four separate growth conditions. The “Uridine” growth condition (uridine supplemented) is the most permissive for cells with mtDNA depletion since mitochondrial respiratory chain deficiency causes pyrimidine auxotrophy (Morais et al, 1982). Media with galactose substituted for glucose (“Galactose”), no uridine supplementation (“No uridine”), or containing low concentration of ethidium bromide (“EtBr”) provides additional stress to cells with mtDNA depletion and may lead to selective loss of *Sucla2*$^{-/-}$ cells with significant mtDNA depletion.

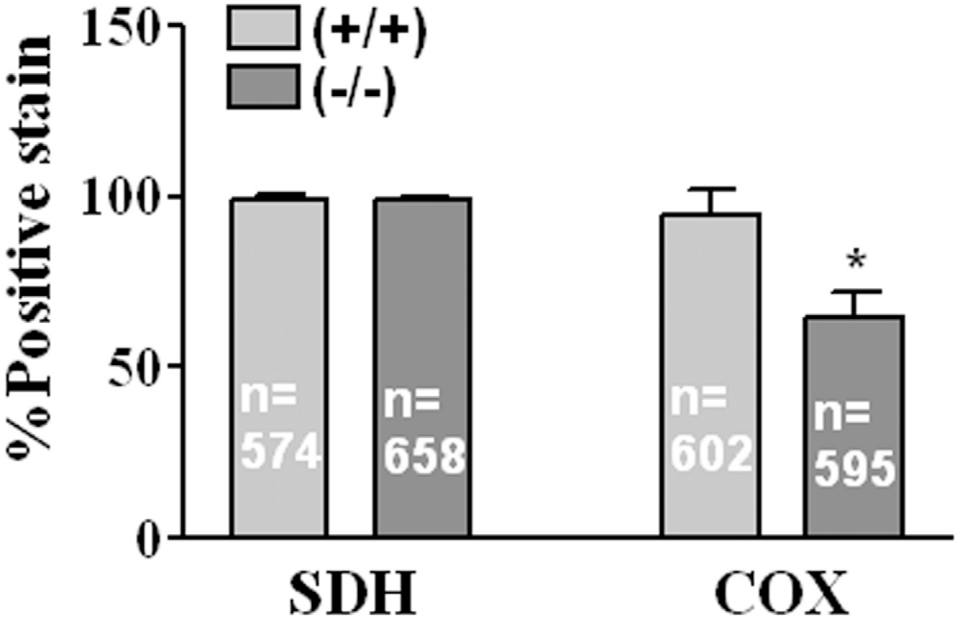

Fig. S4. *Sucla2*$^{-/-}$ cells demonstrate significant loss of COX activity. MEFs that were histochemically stained for succinate dehydrogenase (SDH, no mtDNA-encoded subunits) and cytochrome c oxidase (COX, has mtDNA encoded subunits) activities (Figure 4D) were subsequently manually microscopically analyzed for either being positive or negative for staining; and the percentage of positively stained cells per field was calculated. Both wild type and mutant cells show largely uniform staining for SDH (99.45±0.8 of Wt and 99.14±0.9 of mutant cells), while compared to control, mutant MEFs exhibit a significantly reduced percentage of positive COX staining (94.64±7.4 of Wt and 64.28±7.64 of mutant cells). *$P<0.05$ by Student's *t*-test.

Figure S5

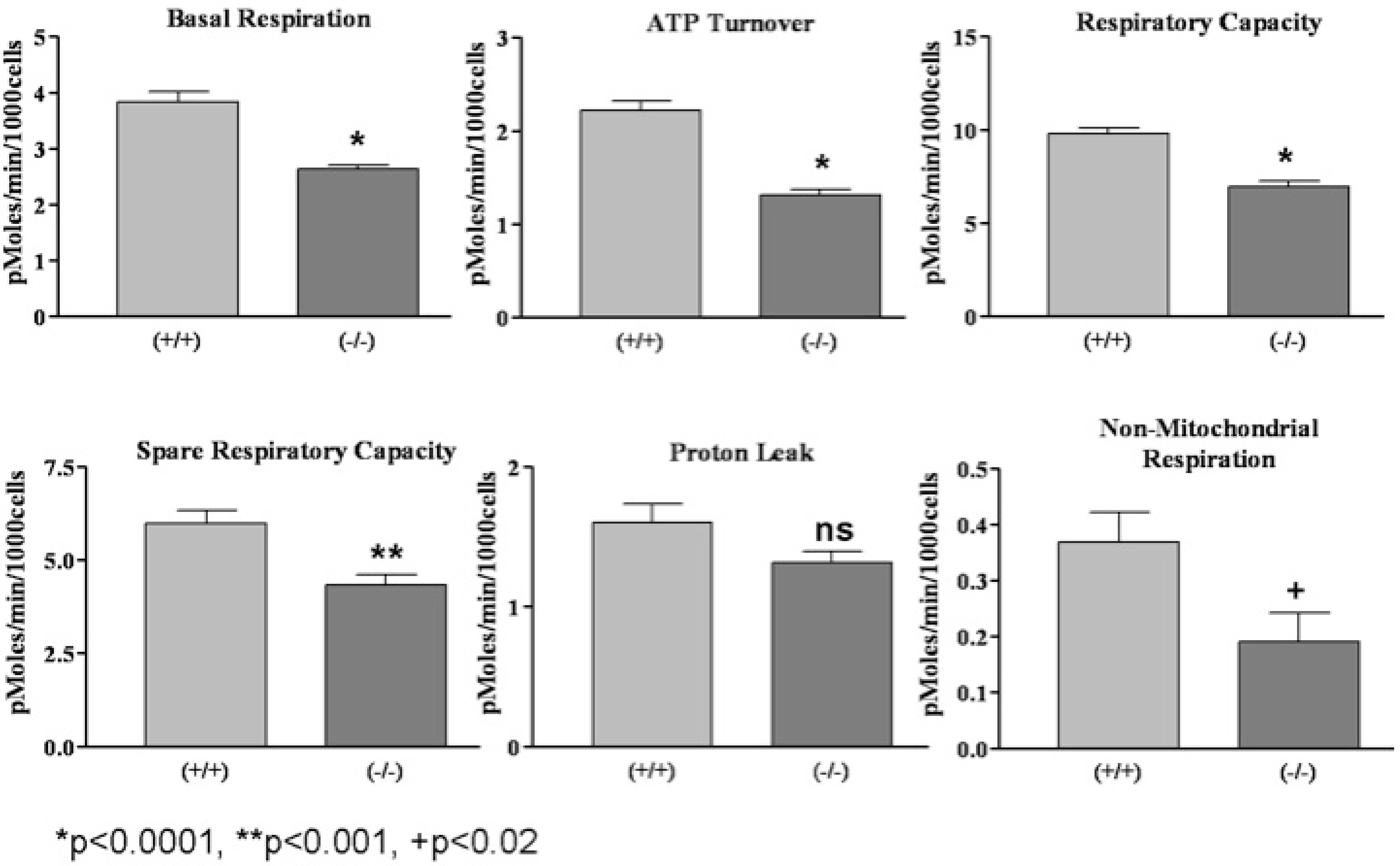

Fig. S5. *Sucla2*$^{-/-}$ cells demonstrate significant respiratory defects. When analyzed for cellular respiration, *Sucla2*$^{-/-}$ cells exhibit functional defects in basal oxygen consumption ("Basal Respiration", oxygen consumption rate (OCR) prior to exposure to oligomycin), oligomycin-sensitive respiration ("ATP Turnover", difference between basal OCR and OCR after exposure to oligomycin), respiratory capacity (difference between FCCP OCR and basal OCR), spare respiratory capacity (difference between FCCP OCR and oligomycin OCR) and non-mitochondrial respiration (difference between OCR after exposure to ETC inhibitors rotenone and antimycin A and background OCR in blank wells). No difference in proton leak (difference between oligomycin OCR and OCR after exposure to ETC inhibitors rotenone and antimycin A) was observed. This data corresponds to the digital tracings depicted in Fig. 4C.

Figure S6

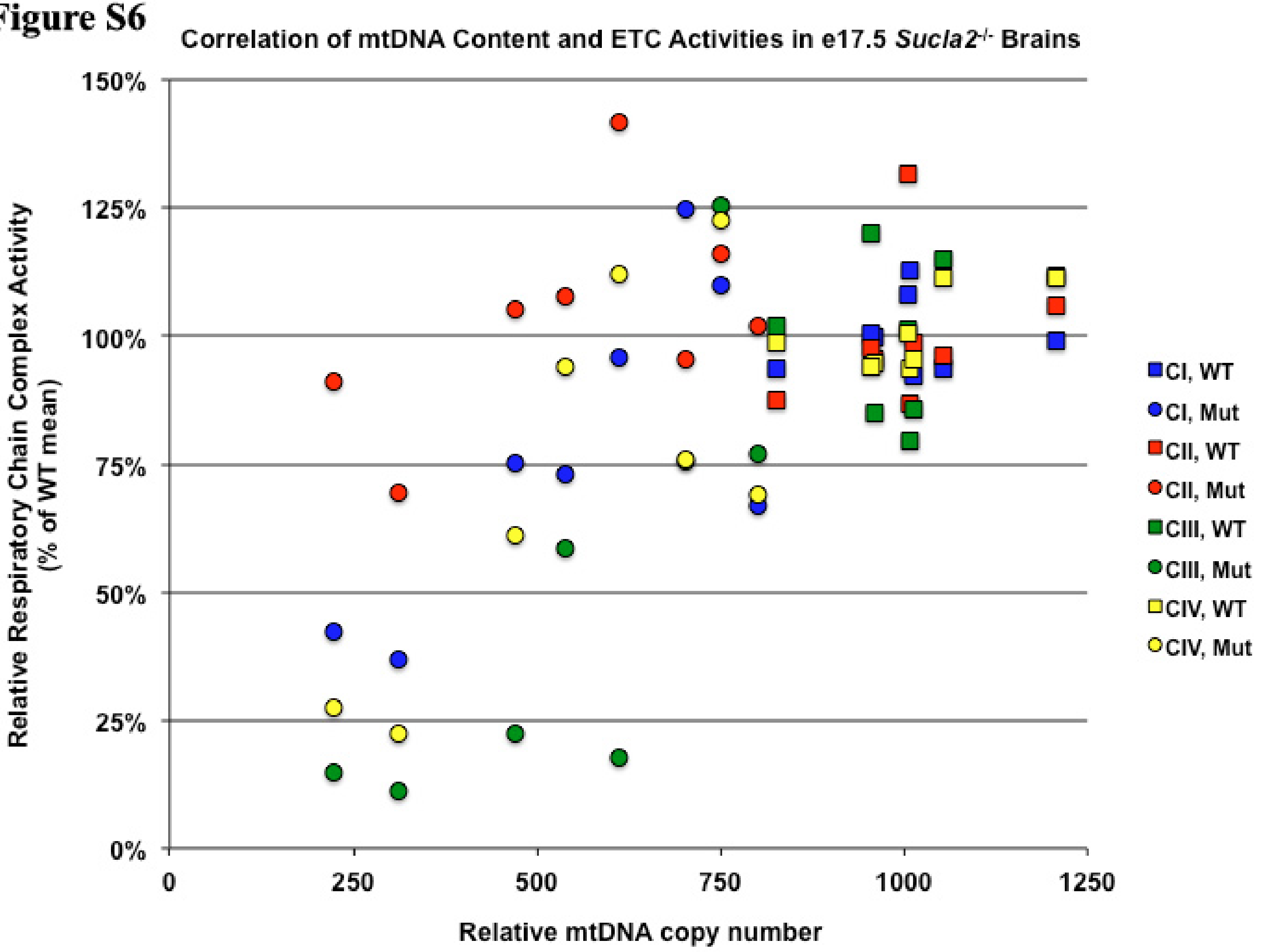

Fig. S6. Mitochondrial respiratory chain deficiency is proportional to mtDNA content in *Sucla2*$^{-/-}$ brain. Brains were dissected from 8 wild type and 8 mutant e17.5 embryos and used for analysis of mitochondrial ETC enzyme activities (complexes I-IV) and for relative mtDNA content. Graph depicts relationship of ETC enzyme activities and mtDNA content. Mutant brains with relative mtDNA copy number below ~600 exhibit proportional loss of enzyme activities for complexes I, III and IV with relative sparing of complex II (the only complex with no mtDNA encoded subunits).

Table S1. Dataset table of identified gene trap mouse ES cell clones
Download Table S1

Table S2. Succinyl-CoA ligase activities from *Sucla2* MEFs
Table S3. Fold change of SCS subunit transcript levels by real-time PCR
Table S4. Relative mtDNA copy number for Sucla2 MEFs, placenta and embryonic tissues
Table S5. Mitochondrial respiratory chain enzyme activities from *Sucla2* MEFs and brain tissue

Table S2. Succinyl coA ligase Activities *Sucla2* MEFs

	ADP/GDP -			ADP+			GDP+			Total activity			ADP/Total		
**Genotype**	**Mean**	**SD**	**N**	**Mean**	**SD**	**N**	**Mean**	**SD**	**N**	**Mean**	**SD**	**N**	**Mean**	**SD**	**N**
Sucla2$^{+/+}$	58.97	0.57	6	63.52	0.56	6	64.04	0.6	6	123.34	1.91	6	0.47	0.01	6
Sucla2$^{-/-}$	58.96	1.03	6	60.29	1.07	6	68.16	1.06	6	119.25	2.1	6	0.13	0.01	6
Sucla2$^{-/-}$***(+R)***	65.19	2.2	6	78.09	1.1	6	78.39	1.9	6	127.64	1.2	6	0.42	0.02	6

Succinyl coA ligase activity expressed as nmol/min/mg protein

Table S3. Fold change (FC) of SCS Subunit Transcript Levels by Real-Time PCR

**Gene**	**Mean FC**	**SD**	**N**
Suclg1	0.98	0.21	3
Sucla2	-5.1	0.32	3
Suclg2	1.1	0.19	3

Table S4. Relative mtDNA copy number for *Sucla2* MEFs, placenta and embryonic tissues

Table S4-A. mtDNA copy#/haploid genome of MEFs from Wild Type and *Sucla2* mutants

	MEFs		
**Genotype**	**Mean**	**SD**	**N**
Sucla2 $^{+/+}$	1199	44	3
Sucla2 $^{+/-}$	1072	50	3
Sucla2 $^{-/-}$	591	178	3

Table S4-B. mtDNA copy#/haploid genome of various tissues from e17.5d Wild Type and *Sucla2* mutants

	Placenta			Brain			Heart			Liver			Muscle		
**Genotype**	**Mean**	**SD**	**N**	**Mean**	**SD**	**N**	**Mean**	**SD**	**N**	**Mean**	**SD**	**N**	**Mean**	**SD**	**N**
Sucla2 $^{+/+}$	1021	107	5	977	80	6	1936	538	6	548	160	6	994	201	6
Sucla2 $^{+/-}$	943	162	16	1133	157	6	2492	619	6	576	314	6	960	113	6
Sucla2 $^{-/-}$	588	48	3	493	221	10	1367	738	10	520	161	10	434	177	10

Table S4-C. mtDNA copy#/haploid genome of various tissues from e15.5d Wild Type and *Sucla2* mutants

	Placenta			Brain			Heart			Liver			Muscle		
**Genotype**	**Mean**	**SD**	**N**	**Mean**	**SD**	**N**	**Mean**	**SD**	**N**	**Mean**	**SD**	**N**	**Mean**	**SD**	**N**
Sucla2 $^{+/+}$	956	177	3	428	87	3	2252	242	3	349	103	3	618	14	3
Sucla2 $^{+/-}$	921	99	8	919	366	3	2269	753	3	798	316	3	863	283	3
Sucla2 $^{-/-}$	798	248	3	1067	175	3	2985	2199	3	620	141	3	925	172	3

Table S5. Mitochondrial respiratory chain enzyme activities from *Sucla2* MEFs and brain tissue

Table S5-A. Respiratory Chain Enzyme Activities of MEF whole cell lysates from Wild Type and *Sucla2* mutants

	Complex I[a]			Complex II[b]			Complex III[c]			Complex IV[d]			Citrate Synthase[e]		
**Genotype**	**Mean**	**SD**	**N**	**Mean**	**SD**	**N**	**Mean**	**SD**	**N**	**Mean**	**SD**	**N**	**Mean**	**SD**	**N**
Sucla2 $^{+/+}$	107.52	1.98	3	8.93	0.41	3	501.90	51.68	3	11.50	0.14	3	109.37	7.34	3
Sucla2 $^{-/-}$	80.36	15.90	3	8.61	0.53	3	260.13	21.23	3	8.39	1.05	3	74.30	14.41	3

Table S5-B. Respiratory Chain Enzyme Activities from Wild Type and *Sucla2* mutant brain whole cell lysates

	Complex I[a]			Complex II[b]			Complex III[c]			Complex IV[d]			Citrate Synthase[e]		
**Genotype**	**Mean**	**SD**	**N**	**Mean**	**SD**	**N**	**Mean**	**SD**	**N**	**Mean**	**SD**	**N**	**Mean**	**SD**	**N**
Sucla2 $^{+/+}$	136.24	16.43	8	18.27	1.86	8	311.23	37.83	8	16.27	1.02	8	88.99	6.56	8
Sucla2 $^{-/-}$	109.77	33.13	8	20.13	2.08	8	160.32	122.70	8	12.03	4.86	8	96.77	19.03	8

[a]NADH:Ubiquinol Oxidoreductase activity in nmol/min/mg protein
[b]Succinate Dehydrogenase activity in nmol/min/mg protein
[c]Ubiquinol:Cytochrome *c* Oxidoreductase activity in nmol/min/mg protein
[d]Cytochrome *c* Oxidase activity in nmol/min/mg protein
[e]nmol/min/mg protein
